# Risk factors associated with multidrug-resistant tuberculosis (MDR-TB) in a tertiary armed force referral and teaching hospital, Ethiopia

**DOI:** 10.1186/s12879-018-3167-9

**Published:** 2018-05-31

**Authors:** Biresaw Demile, Amare Zenebu, Haile Shewaye, Siqing Xia, Awoke Guadie

**Affiliations:** 1 Department of Ophthalmology, Shanghai Tenth People’s Hospital, Tongji University, School of Medicine, Shanghai, China; 2 Department of TB/HIV, Armed Force Referral and Teaching Hospital, Addis Ababa, Ethiopia; 3 State Key Laboratory of Pollution Control and Resource Reuse, College of Environmental Science and Engineering, Tongji University, Shanghai, China; 4 Department of Biology, College of Natural Sciences, Arba Minch University, P.O. Box 21, Arba Minch, Ethiopia

**Keywords:** Tuberculosis, Armed force, Ethiopia, Drug susceptibility, Risk factors

## Abstract

**Background:**

Ethiopia is one of the world health organization defined higher tuberculosis (TB) burden countries where the disease remains a massive public health threat. This study aimed to identify the prevalence and associated factors of multidrug-resistant tuberculosis (MDR-TB) using all armed force and civilian TB attendants in a tertiary level armed force hospital, where data for MDR-TB are previously unpublished.

**Methods:**

Cross-sectional study was conducted from September 2014 to August 2015 in a tertiary level Armed Force Referral and Teaching Hospital (AFRTH), Ethiopia. Armed force members (*n* = 251) and civilians (*n* = 130) which has been undergone TB diagnosis at AFRTH were included. All the specimens collected were subjected to microscopic smear observation, culture growth and drug susceptibility testing. Data were analyzed using statistical package for social sciences following binary logistic regression and Chi-square. *P-values* < 0.05 were considered statistically significant.

**Results:**

Among 381 TB patients, 355 (93.2%) new and 26 (6.8%) retreatment cases were identified. Culture and smear positive TB cases were identified in 297 (77.9%) and 252 (66.1%) patients, respectively. The overall prevalence of MDR-TB in AFRTH was found 1.8% (1.3% for armed force members and 0.5% for civilian patients) all of which were previously TB treated cases. The entire treatment success rates were 92.6% achieved highest in the armed force (active and pension) than the civilian patients. The failure and dead cases were also found 2.5 and 4.6%, respectively. Using bivariate analysis, category of attendants and TB contact history were strong predictors of MDR-TB in armed force and civilian patients. Moreover, human immunodeficiency virus (HIV) infection also identified a significant (OR = 14.6; 95% CI = 2.3–92.1; *p* = 0.004) predicting factor for MDR-TB in armed force members. However, sex, age and body mass index were not associated factor for MDR-TB.

**Conclusions:**

In AFRTH, lower prevalence of MDR-TB was identified in armed force and civilian patients that were significantly associated with category of attendants, HIV infection and TB contact history. Considering armed force society as one segment of population significantly helps to plan a better MDR-TB control management, especially for countries classified as TB high burden country.

## Background

Tuberculosis (TB) is an infectious disease caused by *Mycobacterium tuberculosis* (*M. tuberculosis*) complex which usually affects the lung [1]. The bacteria are transmitted via close contact with an infected individual who is actively spreading the bacteria through coughing [2]. Once inhaled, the infection remains latent for decades in 90 to 95% healthy adult [1–3]. However, illness of latent TB manifested only when the bacteria become active. There are many factors that contribute the latent TB bacteria become active including human immunodeficiency virus (HIV), older age, diabetics, close contact with an active case of TB disease and other immunocompromising illness conditions [1, 4].

Although TB is an old disease with many efforts to treat and control, still it remains the main cause of morbidity for millions of people each year [1, 3]. According to World Health Organization (WHO) estimates showed that there were almost 9.6 (5.4 men, 3.2 women and 1.0 children) million new TB cases globally in 2014, of which 1.5 million cases were accounted TB deaths [5]. The same WHO report also showed that 86% of TB infection is from South-East Asia and Western Pacific (58%) and African (28%) regions. The presence of relatively higher HIV patient in these regions significantly contributed an increased incidence of TB [6]. Ethiopia is one of the WHO defined higher TB burden countries where the disease remains a massive public health threat and an economic burden. World Health Organization in 2016 listed Ethiopia 10th out of the 30 high TB priority countries in the globe [1].

Based on WHO recommended six-month standard course of medication, several countries treat TB disease using four first-line (rifampin, isoniazid, pyrazinamide and ethambutol) anti-TB drugs [2, 7, 8]. When *M. tuberculosis* becomes resistant to treatment with at least the two first-line drugs (i.e, isoniazid and rifampin), the condition is known as multidrug-resistant tuberculosis (MDR-TB) [1, 3, 9]. Previous studies mentioned that *M. tuberculosis* develops various drug-resistance mechanisms by using its special cellular structure and metabolic system [9, 10]. For instance, the unique structures like mycolic acid (as part of the cell wall) and trans-membrane protein help the *M. tuberculosis* to restrict entry of drug molecules to the cell, and to pump out antibiotics from the cell, respectively [10, 11]. The *M. tuberculosis* also utilizes different enzymatic strategies to alter the structure of drug synthesis target sites (such as ribosomes and deoxyribonucleic acid) and thereby avoid the action of antibiotics [12]. Moreover, there are also reports that mentioned the ability of *M. tuberculosis* directly modify the anti-TB drug into another form which in turn leads to inactivate the target drug compound action designed for its specific cellular site [10–13]. Inadequate treatment (due to shortage of drug, increasing cost of drug and physician errors) and inadequate adherence (such as poor compliance, alcoholism, drug addiction, length of treatment and adverse drug reactions) have been also identified as a drug resistance enhancing mechanisms by creating a selective pressure for a rapid evolution of *M. tuberculosis* [14–17].

Globally, 3.5% of new and 20.5% of previously treated TB patients were estimated to have had MDR-TB [1]. Sub-Saharan Africa represents 14% of the global burden of new MDR-TB cases [18]. World Health Organization in 2016 listed Ethiopia 8th out of 30 high MDR-TB burden countries in the world with a prevalence of 2.7% (1.5–4.0) in newly and 14.0% (3.6–25.0) in previously treated TB patients. Like Ethiopia which is listed 3rd, other six countries in Africa including (new/retreatment % accordingly) Angola (2.6/18%), DR Congo (2.2/17%), Kenya (1.3/9.4%), Nigeria (4.3/25%), Somalia (8.7/47%) and Zimbabwe (4.6/14%) also listed among the 30 high MDR-TB burden countries in the world [1]. Although MDR-TB is a growing concern in Africa where limited resource exists, it is largely under-reported [18, 19]. In Ethiopia, many of the MDR-TB patients are remain undiagnosed due to the low socioeconomic status of the population, lack of awareness and inaccessibility of health service. For instance, WHO in 2012 estimated that the number of patients in Ethiopia tested for MDR-TB was < 1% of new and < 4% of retreatment cases [5].

There are small numbers of MDR-TB studies in different regions of Ethiopia [16, 20–22], however, most of these surveys were restricted only to civilian patients and civilian hospitals. To the best of our knowledge, there is no published information about the status of TB and MDR-TB concerning armed force as one segment of the population in Ethiopia. This condition significantly compromises the MDR-TB control efforts. Therefore, this study has been designed to evaluate the prevalence and risk factors of MDR-TB using armed force and civilian patients in a tertiary level Armed Force Referral and Teaching Hospital (AFRTH) Addis Ababa, Ethiopia. The subjects were from Ministry of Defense members (active military and pension) and civilian clients which have got service from AFRTH.

## Methods

### Study area

The study was conducted at AFRTH which is located in Addis Ababa, the capital city of Ethiopia. It is the only referral and teaching military hospital of the country at the rank of tertiary level. It is organized under Health Main Directorate (HMD), Ministry of Defense.

Armed Force Referral and Teaching Hospital provides medical services to members of the Ethiopian defense forces. It accepts referral case from all secondary level (Eastern, Central, North and South-western) command referral hospitals located all over the country. In AFRTH, there is also a limited ward allocated to give service to civilian clients. Although the AFRTH provides service for a large number of TB patients per year, however, the data included in this study were only collected from those patients who had complete information registered according to National Tuberculosis and Leprosy Control Program (NTLCP) guideline of Ethiopia [7] adopted from WHO.

### Definition of TB cases and treatment outcome

According to the standard definitions of NTLCP guideline of Ethiopia [7], the following case and treatment outcome definitions were used in the current study. The following are case definitions: (i) new case is used if a patient who never had treatment for TB or has been on previous anti-TB treatment for less than 4 wk in the past, (ii) relapse if a patient declared cured or whose treatment was completed of any form of TB in the past, but who reports back to the health service and is now found to be microscopic smear positive or culture positive, (iii) treatment failure if a patient who is smear positive at the end of the fifth month or later, after commencing and it also includes a patient who was initially sputum smear negative but who becomes smear positive during treatment, (iv) return after default if a patient previously recorded as defaulted from treatment and returns to the health facility with smear positive sputum, and (v) others if a patient who does not fit in any of the above mentioned categories (e.g, smear negative pulmonary TB case who returns after default, extaplumonary TB case returning after default and previously treated TB patients with an unknown outcome of that previous treatment).

According to NTLCP guideline [7], the following treatment outcome definitions were also used in the current study: (i) cured if patients have finished treatment with negative bacteriology result at the end of treatment, (ii) treatment completed if the patient finished treatment, but without bacteriology result at the end of treatment, (iii) treatment failure if a TB patient remains smear positive at 5 mo follow-up despite correct intake of medication, (iv) defaulted treatment if the patients who interrupted their treatment for two consecutive months or more than 2 mo after registration, (v) died if the patient died from any cause during the course of treatment, (vi) transfer out if the patient treatment result is unknown due to transfer to another health facility, and (vi) treatment success is used defined as the sum of cured and completed treatments.

### Study design, data collection and laboratory sputum sample processing

A cross-sectional study was conducted following the most recent WHO guidelines for surveillance of drug-resistance in TB [23]. In this study, all patients who diagnosed TB in AFRTH between September 2014 to August 2015 and whose information found to be complete and qualify the NTLCP guidelines had been included. The study subjects include direct register patients at AFRTH (armed force members and civilians) and those referred from secondary level armed hospitals (armed force members only) located all over the country. As a result, our study population especially the armed force members appears to be a representative sample of all military members at risk for TB that are located all over Ethiopia.

In AFRTH, the suspected TB patients were first identified through examining signs and symptoms, chest x-ray and prior history of TB. Although a large number of patients had been registered in the AFRTH-TB Clinic, those who failed to qualify the preliminary examination have not been included in the current study. Among 389 cases qualified for preliminary examination, 8 suspected TB cases were also excluded due to incomplete information.

Demographic data such as gender, age, HIV status, the category of attendants and TB contact history were collected from the patient record books. In addition, routine data were obtained from AFRTH laboratory reports for sputum smear microscopy, culture growth and drug susceptibility testing (DST). The clinical samples were processed using the N-acetyl-L-cysteine NaOH (NALC-NaOH) method. The processed samples were suspended in 1000 mL neutral sterile phosphate buffer and then 100 mL of resuspended pellet was inoculated onto Lowenstein–Jensen (LJ) medium slants. *M. tuberculosis* was confirmed in cultures using measurements of growth rate, colony morphology, pigmentation and commercial biochemical tests. The biochemical testing (niacin assay, nitrate reduction, and catalase tests) was used to identify the isolated *Mycobacterium* once they were categorized into a preliminary subgroup based on their growth characteristics [24]. All isolates of *M. tuberculosis* were subjected to DST using concentration method. Drug susceptibility testing for the four (rifampin, isoniazid, streptomycin and ethambutol) first-line anti-TB drug was performed according to WHO guidelines [23]. Smear for microscopic examination was also stained using the Ziehl–Neelsen method and results were reported as smear positive and negative.

The AFRTH laboratory was subjected to quality control through WHO guideline [23]. Drug susceptibility testing quality control was done using standard strains of *M. tuberculosis* (H37Rv). All data included here were reported by qualified laboratory technicians and physicians.

### Statistical analysis

The data were analyzed using Epi Info 6, Excel 2010 and statistical package for the social sciences (SPSS) version 20.0 (IBM, NY, USA). A total of 389 patient information’s were initially enter into the data system, however, eight (2.0%) patients whose information were found to be incomplete and excluded during data analysis. Either binary logistic regression or Chi-square (likelihood ratio) statistics were used to assess the possible risk factors associated with the dependent variable MDR-TB and the independent variables such as sex, age, body mass index, patient categories, TB contact history and HIV infection. Two-sided *p-*values were considered significant when the value was less than 0.05.

## Results

### Demography, culture and drug susceptibility tests

As shown in Table 1, a total of 389 clinically diagnosed TB patients were enrolled in AFRTH, of which 98% cases were included in this study. Eight (2.0%) cases were excluded for MTB culture growth, microscopic smear observation and drug sensitivity tests due to incomplete demography data (*n* = 5) and contamination (*n* = 3). The new and retreatment TB cases included were found 355 (93.2%) and 26 (6.8%), respectively. Based on the category of patients registered in AFRTH, active armed force, pension and civilians were found 216 (56.7%), 35 (9.2) and 130 (34.1%), respectively.

**Table 1:** Classification of cases included from new and TB-retreatment cases

Classification	Total-patients		TB-new patients		TB-previously treated patients	
	Number	%	Number	%	Number	%
Total TB-patients	389	100	362	100	27	100
TB-patients excluded	8	2.0	7	1.9	1	3.7
TB-patients included	381	98.0	355	98.1	26	96.3
Category of attendants						
Armed force-Active	216	56.7	203	57.2	13	50.0
Armed force-Pension	35	9.2	26	7.3	9	34.6
Civil	130	34.1	126	35.5	4	15.4

As shown in Table 2, the proportion of men and women patients were found 273 (71.7%) and 108 (28.3%), respectively resulting male to female ratio to be 2.5 to 1. The age of the study participants varies from 18 to 96 years with a median age of 34 years. The mean age of the patients was 36.76 ± 13.84 years, of which 288 (75.6%) patients were in the age range of 18–45 years (most in the active armed force).

**Table 2:** General demographic and clinical findings of civilian and armed force patients in AFRTH

Variables	Total cases (*n* = 381)		Civilian (*n* = 130)		Armed force members			
					Active		Pension	
					(*n* = 216)		(*n* = 35)	
	No	%	No	%	No	%	No	%
Sex								
Female	108	28.3	63	48.5	42	19.4	4	11.4
Male	273	71.7	67	51.5	174	80.6	31	88.6
Age (year)								
18–44	288	75.6	96	73.8	192	88.9	0	0
≥ 45	93	24.4	34	26.2	24	11.1	35	100
Body mass index (kg/m^2^)								
< 18.5	39	10.2	15	11.5	22	10.2	2	5.7
18.5–25	217	57.0	67	51.5	129	59.7	21	60.0
> 25	125	32.8	48	37.0	65	30.1	12	34.3
History of TB contact								
Yes	26	6.8	6	4.6	15	6.9	5	14.3
No	355	93.2	124	95.4	201	93.1	30	85.7
Culture growth on LJ media								
Positive	297	77.9	93	71.5	170	78.7	34	97.1
Negative	84	22.1	37	28.5	46	21.3	1	2.9
Microscopic smear observation								
Positive	252	66.1	90	69.2	139	64.4	23	65.7
Negative	129	33.9	40	30.8	77	35.6	12	34.3
Drug susceptibility testing								
MDR-TB	7	1.8	2	1.5	2	0.9	3	8.6
Non-MDR-TB	374	98.2	128	98.5	214	99.1	32	91.4
TB-patient categories at start								
New	355	93.2	126	97.0	203	94.0	26	74.2
Relapse	6	1.6	2	1.5	7	3.2	7	20.0
Failure	1	0.3	0	0.0	1	0.5	0	0.0
Return after default	2	0.6	0	0.0	1	0.5	1	2.9
Others	17	4.3	2	1.5	4	1.8	1	2.9
TB-treatment outcome								
Complete	216	56.7	69	53.1	121	56.0	26	74.3
Cure	48	12.6	5	3.8	41	19.0	2	5.7
Dead	13	3.4	2	1.5	11	5.1	0	0
Failure	7	1.8	1	0.8	1	0.5	5	14.3
Transfer out	97	25.5	53	40.8	42	19.4	2	5.7
HIV result								
Positive	34	8.9	9	6.9	15	7.0	10	28.6
Negative	321	84.3	105	80.8	191	88.4	25	71.4
Not tested	26	6.8	16	12.3	10	4.6	0	0
Antiretroviral therapy status								
ART started	28	7.4	8	6.1	12	5.6	8	22.9
ART not started	6	1.6	1	0.8	3	1.4	2	5.7
HIV negative	321	84.2	105	80.8	191	88.4	25	71.4
HIV not tested	26	6.8	16	12.3	10	4.6	0	0

Among 381 TB suspected patients, 26 (6.8%) patients have TB contact history, particularly attendants from the armed force was higher (*n* = 20). Culture growth on LJ media was observed from 297 (77.9%) specimens. The pension sputum samples almost all (97.1%) found culture positive. However, in the active armed force and civilians specimens, the culture positive results were below 80%. Compared with culture positive samples, overall 66.1% (*n* = 252) of microscopic smear TB positive samples were identified which has been relatively lower in all patient groups (69.2% for civilian, 64.4% for active and 65.7% for pension armed force members) than the corresponding culture growth results. The DST results showed that 7(1.8%) specimens identified as MDR-TB cases and 374 (98.2%) specimens non-MDR-TB cases. The number of civilian and armed force members with MDR-TB was identified in 2 (0.5%) and 5 (1.3%) patients, respectively in which all patients were previously treated TB case (Table 2). There were no new MDR-TB cases identified, rather all MDR-TB patients have been found to be a failure (*n* = 1) and relapse (*n* = 6) cases (Table 2).

In AFRTH, all smear positive (*n* = 252) and negative (*n* = 129) patients were treated with first-line TB drugs. Except for 97 (25.5%) cases that have been transferred out (results unknown), most (74.5%) TB patient outcomes were known. Most of the TB patients completed (56.7%) the treatment, while 12.6, 3.4 and 1.8% were a cure, dead and failure cases, respectively. All the dead cases (*n* = 13) were newly diagnosed patients which were identified from active armed force members (*n* = 11) and civilian patients (n = 2). Interestingly, in the current study there was no defaulter treatment outcome seen either in civilian or armed force patients (Table 2). Compared with Ethiopian national TB treatment success rate (84.0%) [1], the current study treatment success rate (69.3%) has been found lower due to most of the transfer out cases (25.5%), which is expected from AFRTH that accepts referred patients from all secondary level (Eastern, Central, North and South-western) command referral hospitals located all over the country.

Although all (*n* = 381) TB patients requested to give blood for HIV test, most (*n* = 355) accepted the offer, particularly all the pension attendants (Table 2). However, 16 (4.2%) civilian patients and 10 (2.6%) active armed force members rejected the offer. Among 114 civilians, 206 active and 35 pension TB patients who accepted the HIV test, respectively 9, 15 and 10 samples were identified HIV positive. Among HIV test positive (*n* = 34) TB patients, 28 (82.4%) patients started taking antiretroviral therapy (ART). However, one civilian, three active military and two pension attendants didn’t start the ART drug (Table 2).

### Multidrug-resistant tuberculosis risk factors

The relationship between individual exposure variables (category of attendants, gender, age, body mass index, TB contact history and HIV status) and the dependent variables (MDR-TB status) were shown in Tables 3 and 4. In general, the category of attendants was the predicting factor (*p* = 0.029) for MDR-TB in AFRTH patients (Table 3). Compared with active armed force members (2/216 = 0.9%) and civilian attendants (2/130 = 1.5%), pensions have shown significant (*p* = 0.013) higher (3/35 = 8.6%) value of MDR-TB positive results. The occurrence of lower MDR-TB positive than MDR-TB negative results was also found a strong predicting factor (*p* = 0.007) for the category of attendants.

**Table 3:** Association of MDR-TB value in armed force and civilian TB patients in AFRTH

Category (*p* = 0.029)	Total	MDR-TB		OR (95% CI)	*p*-value
		+	–		
Active-Armed force	216	2	214	Ref	Ref
Pension-Armed force	35	3	32	10.0 (1.6–62.4)	0.013
Civilian	130	2	128	1.7 (0.2–12.0)	0.610
*p*-value	–	0.007 (χ^2^ = 9.86)		–	–

*OR* Odds ratio, *CI* Confident interval, *Ref* Reference for binary logistic value, *MDR-TB* Multidrug resistant tuberculosis

+ Positive, − Negative, *χ*^*2*^ Chi-square

**Table 4:** Factors associated with MDR-TB in AFRTH patients (armed force and civilian)

Variables	Armed force members				Civilian			
	MDR-TB		OR (95% CI)	*p-*value	MDR-TB		OR (95% CI)	*p-*value
	+	–			+	–		
Sex								
Female	1	45	1.0 (1.0–1.1)	0.153	1	62	1.1 (0.1–17.4)	0.965
Male	4	201	Ref	Ref	1	66	Ref	Ref
Age (year)								
18–44	2	190	Ref	Ref	1	90	Ref	Ref
≥ 45	3	56	5.1 (0.8–31.2)	0.079	1	38	2.4 (0.14–38.9)	0.546
Body mass index (kg/m^2^)								
< 18.5	2	22	6.9 (0.6–79.8)	0.122	1	15	3.4 (0.2–57.2)	0.402
18.5–25	2	148	1.0 (0.9–11.5)	0.983	0	67	0.6 (0.1–4.5)	0.581
> 25	1	76	Ref	Ref	1	48	Ref	Ref
History of TB contact								
No	1	19	Ref	Ref	1	123	Ref	Ref
Yes	4	227	57.5 (6.1–545.1)	0.0004	1	5	0.04 (0.02–0.8)	0.031
HIV result^a^								
Negative	2	214	Ref	Ref	1	104	Ref	
Positive	3	22	14.6 (2.3–92.1)	0.004	1	8	13.0 (0.7–227.8)	0.079

*OR* Odds ratio, *CI* Confident interval, *Ref* Reference

+ Positive, − Negative

^a^16 from civil and 10 from armed force excluded

As shown in Table 4, HIV infection result (OR = 14.6; 95% CI = 2.3–92.1; *p* = 0.004) and TB contact history (OR = 57.5; 95% CI = 6.1–545.1, *p* = 0.0004) were significantly associated with MDR-TB in armed force members. However, sex (OR = 1.0; 95% CI = 1.0–1.1; *p* = 0.153), age (OR = 5.1; 95% CI = 0.8–31.2; *p* = 0.079) and body mass index (OR = 1.0–6.9; 95% CI = 0.6–79.8; *p* = 0.122–0.983) were not the predicting factor for MDR-TB in armed force members.

In civilian patients, TB contact history has been found strong predicting factor for MDR-TB (OR = 0.04, 95% CI = 0.02–0.8; *p* = 0.031). However, sex (OR = 1.1; 95% CI = 0.1–17.4; *p* = 0.965), age (OR = 2.4; 95% CI = 0.14–38.9; *p* = 0.546), body mass index (OR = 0.6–3.4; 95% CI = 0.1–57.2; *p* = 0.402–0.581) and HIV infection (OR = 13.0; 95% CI = 0.7–227.8; *p* = 0.079) were not associated with MDR-TB in civilian patients.

## Discussion

TB remains the major global health problem which ranked the 9th leading cause of death worldwide [1]. Currently, the emergency of MDR-TB is also the main public health problem in both developing and developed countries. Globally, the prevalence of MDR-TB case among the newly and previously treated TB patients has been found 3.5 and 20.5%, respectively [1]. The same WHO report also indicated that 7 African countries are listed out of 30 high burden MD-RTB countries in the world with the overall prevalence of 2.7% new and 14.0% previously treated cases [1].

In the current study, the overall prevalence of MDR-TB in armed force and civilian patients were identified 1.8%, in which all have been found previously TB treated patients. Although published data is deficient to compare this study with a similar setting (armed force + civilian), there is limited information reported in some countries focus on armed force patients (Table 5). Most of the studies found in the literature were civilian patients carried out in civilian hospital. The prevalence reports were in agreement with our study, particularly studies conducted in Indian (1.2%) and Turkey (2.7%) that focus on armed force patients (Table 5). A study on USA military population also stated that the incidence of TB disease identified in military population has been found eight times lower (0.4 per 100,000) than the overall USA population (3.0 per 100,000) which supports the current study recorded lower MDR-TB infection [2]. Compared with the current study, relatively higher MDR-TB prevalence result also reported in Korean young armed force patients (Table 5). Compared with Ethiopian overall MDR-TB reported data (2.7% in newly and 14.0% in previously treated patients), the current MDR-TB prevalence result observed in AFRH is also found much smaller [1].

**Table 5:** Comparing MDR-TB in this study with other previous findings

Study area	Study population	Sample size (Number)	MDR-TB prevalence (%)	Reference
TH in Addis Ababa, Ethiopia	Armed force members + civilian	381	1.8	This study
Chest hospital in Istanbul, Turkey	Armed force members	365	2.7	[32]
Northwest, India	Armed force members	172	1.2	[27]
Tertiary chest hospital, India	Armed force members	1120	4.2	[30]
AF capital hospital, Korea	Armed force members (young)	198	8.1	[8]
Eastern, Ethiopia	Civilian	357	1.1	[16]
Northwest, Ethiopia	Civilian	124	5.7	[21]
Northeast, China	Civilian	205	6.8	[33]
Southeast, Nigeria	Civilian	180	7.7	[34]
Harare, Zimbabwe	Civilian	213	12.0	[28]
Sinaloa, Mexico	Civilian	671	17.9	[35]
Four regions, Swaziland	Civilian	633	19.3	[29]
Four sentinel sites, Georgia	Civilian	931	28.1	[36]
Kassala, Sudan	Civilian	60	30.0	[37]
Oromia region, Ethiopia	Civilian	265	33.2	[22]
Amara region, Ethiopia	Civilian	413	36.3	[20]
Samara region, Russia	Civilian + prisoner	600	45.5	[38]

*TH* Tertiary hospital, *AF* Armed force, *AFRTH* Armed force referral and teaching hospital

Except Seyoum et al. [16] reported lower (1.1%) MDR-TB prevalence, several other studies carried out in Ethiopia using civilians as a study participant was reported a higher result of MDR-TB results (Table 5). For instance, using civilian patients the prevalence of MDR-TB was 36.3% in Amara [20] and 33.2% in Oromia Regions [22] of Ethiopia. Compared with other countries study on MDR-TB prevalence using civilian society (Table 5), the current study also much lower than reports from Georgia (28.1%), Eastern Sudan (30.0%), Swaziland (19.3%), Zimbabwe (12.0%), Samara in Russia (45.5%) and Sinaloa in Mexico (17.9%).

The lower MDR-TB prevalence reported in this study primarily due to the variations in the selection of patient groups studied. In the present study, the subjects were civilian and armed force members. The armed force members were non-referred (living in Addis Ababa) and referred cases from various secondary command referral hospitals which are located in the different geographical location of the country. This makes the current study unique from surveys carried out by other workers mostly covering a particular geographical region using civilian subjects. The variation might also due to sample size, time of the study, access to health care facilities, and effectiveness of TB control programs. Compared to a remote area of Ethiopian health centers which deal with civilian population, there is an effective functioning of TB control program in military societies. The regular supplies of anti-tuberculosis drugs, well-organized patient diagnosis, treatment follow-up and good patient adherence are effectively implemented in armed forces which presumably contribute to the lower prevalence of MDR-TB in the current study. Indeed, this has been well reflected in the patient categories which include the civilian, pension and active armed members (Table 3).

With analysis of binary logistic regression model, the category of attendants showed a statistically significant difference with MDR-TB, particularly pension attendants were ten times more likely at risk for MDR-TB (OR = 10.0; 95% CI = 1.6–62.40; *p* = 0.013) than active armed force members. However, the active military attendants and civilian patients didn’t show statistically significant (*p* = 0.610) difference for MDR-TB positive result. Although it needs further investigation, the insignificant variation among active armed force and civilian attendants suggested that the later went to a tertiary level AFRTH for seeking better medical service that has been offered for limited private wing clients most probably in a better economic status. Previous studies showed that annual income status has been found a significant risk factor for MDR-TB prevalence [15, 25]. The statistical significance difference observed among pension and active military members were also most probably due to living environment/lifestyle and age of the patients. For example, health education (one time/week) and sanitation (two times/week) programs have been designed and implemented in active military societies that might help to reduce *M. tuberculosis* infection that aggravated due to poor hygiene and ventilation [15, 25]*.* Moreover, the early treatment made in active military society without any cost from the patient side might probably contribute to reducing the spread of drug-resistant TB in the community [26]. It is also clear that pension attendant is expected to have a high probability of developing MDR-TB than active military staff which is related to advancement in age [1]. Although not statistically significant (*p* = 0.079 and 0.546), advancement in age has been found 5 and 2 times more likely at risk for MDR-TB than younger age in armed force and civilian patients, respectively (Table 4). Moreover, pension attendants’ loss most of the active military privileges (such as health education, sanitation, early treatment and follow-up) which might enhance the probability of pension attendants contracted with TB bacteria that resist drug [11, 15, 25].

In this study, history of contact with TB patient has been found the predicting factor of MDR-TB for both armed force members (*p* = 0.0004) and civilian (*p* = 0.031) patients. When all cases (*n* = 381) merged together and analyzed, pension (OR = 3.4; 95% CI = 1.0–12.1) and civilian attendant (OR = 1.5; 95% CI = 0.6–4.1) have a much higher risk of TB infected person contact than the active military staff which presumably suggested that the TB control is better managed in active military society through regular education about communicable and non-communicable diseases, and sanitation programs. Of course, *M. tuberculosis* is transmitted via close contact with an infected individual who is actively spreading the bacteria through coughing [1, 2]. Once inhaled, the infection is established with or without a visible primary lung lesion; lymphatic and hematogenous spread usually follows within 3 weeks of infection [2]. This study is in agreement with the study in USA military that mentioned higher TB risk among service members who may be exposed to infected persons, such as personnel involved in humanitarian assistance and health care operations serving local, high-risk populations [2].

Among 381 TB suspected patients, the new and retreatment TB cases that showed growth on the media were found 71.1 and 6.8%, respectively. However, 11.8% of culture positive samples did not found smear positive. The growth of *M. tuberculosis* on LJ media in cases of smear negative for acid-fast bacilli is a known phenomenon as 10^5^ bacilli per mL of sputum are required for the organism to be seen on light microscope but culture may show growth [27].

In the current study, the number of HIV positive and negative patients were identified 34 (9.6%) and 321 (90.4%) for all patients tested, respectively. Compared with other studies [28, 29] in Swaziland (22.6% = 102/451) and Zimbabwe (74.0% = 157/211), HIV positive patients are relatively lower in this study (25/241 in armed force members and 9/114 in civilian patients). However, the HIV positive results were found a significant predicting factor for MDR-TB (OR = 14.6; 95% CI = 2.3–92.1; *p* = 0.004) in armed force members. Particularly, the infection is magnificent in pension attendants (10 out of 25) (Table 3), suggested that during the study period the pension staff with HIV might frequently register at AFRTH for medical service which is provided free as a staff member. Moreover, pension staffs are at the older age in which much of the physiological activities are downgraded and contributed to the co-infection of HIV-TB under immunocompromised conditions [28, 29]. Statistical analysis also showed that there was a positive significant correlation between MDR-TB and HIV co-infection (*r* = 0.229; *p* < 0.01).

In the current study, the status of 284 (74.5%) patients was identified while 97 (25.5%) patients transferred out and status unknown. Among the status identified patients, the treatment success rate was found 93.0%, highest in active armed force followed by pension and civilian patients (Table 2). The higher rate of treatment success in armed force patients most probably indicates that there is a good efficacy of the standard treatment regimen in armed force society. The adequate follow-up, early identification and management of adverse drug reactions had been mentioned the key to favorable treatment outcome success [26, 30]. Drug sensitive pulmonary TB is generally treated with four active drugs isoniazid, rifampin, pyrazinamide and ethambutol [17]. These drugs are continued for the first 2 months of therapy and are subsequently followed by at least 4 mo of two drugs (most commonly with isoniazid and rifampin). There was no default cases observed in the current study. However, 7.0% treatment failure rate was attributed due to death and failure cases. Similar to this study, 9.0% failure rate (dead and failure) was recorded in Germany [31]. However, higher treatment failure rate was observed in other studies in civilian hospitals [20, 26, 29], suggesting that TB drug administration in AFRTH is implemented efficiently.

Our retrospective study has limitations. Since reports were not designed for study purposes, some demographic MDR-TB predicting factors such as annual income, size of living space, family history, history of prison and others were lacking. The clinical treatment outcome of referred outpatients to secondary level command hospitals was not identified. Follow-up time was recorded to the completion of treatment at AFRTH. Although this time frame is sufficient for documenting surveillance-based treatment outcomes, it may not be sufficient to assess long-term clinical outcomes. It was also difficult to distinguish the referred patient where they came (which military command hospital) that might help to identify which geographical location contributed more to MDR-TB case and plan better management. Despite these limitations, the current study provides information about the MDR-TB prevalence and associated factors in AFRTH where data has not been previously published. Moreover, comparative studies among armed force members (active and pension) and civilian patients also unique to this study to provide information about MDR-TB.

## Conclusions

MDR-TB is a major public health problem and mainly affects economically productive age group of the population. Compared with many civilian hospitals, the prevalence of MDR-TB in AFRTH was found low. This shows that TB control management is well implemented in armed society. In general, this study might be scale-up and applicable all over in Ethiopia and elsewhere in the world, where information is scant (armed force society) to improve the availability and quality of MDR-TB service.

## Abbreviations

AF: Armed force
AFRTH: Armed force referral and teaching hospital
ART: Antiretroviral therapy
CI: Confidence interval
DST: Drug susceptibility testing
HIV: Human immunodeficiency virus
HMD: Health main directorate
LJ: Lowenstein Jensen
MDR-TB: Multidrug-resistant tuberculosis
OR: Odds ratio
Ref: Reference
SPSS: Statistical package for the social sciences
TB: Tuberculosis
TH: Tertiary hospital
WHO: World Health Organization
